# Diabetic kidney disease: new clinical and therapeutic issues. Joint position statement of the Italian Diabetes Society and the Italian Society of Nephrology on “The natural history of diabetic kidney disease and treatment of hyperglycemia in patients with type 2 diabetes and impaired renal function”

**DOI:** 10.1007/s40620-019-00650-x

**Published:** 2019-10-02

**Authors:** Giuseppe Pugliese, Giuseppe Penno, Andrea Natali, Federica Barutta, Salvatore Di Paolo, Gianpaolo Reboldi, Loreto Gesualdo, Luca De Nicola, the Italian Diabetes Society and the Italian Society of Nephrology

**Affiliations:** 1 Department of Clinical and Molecular Medicine, “La Sapienza” University, Rome, Italy; 2 Endocrine and Metabolic Unit, Sant’Andrea University Hospital, Rome, Italy; 3 Department of Clinical and Experimental Medicine, University of Pisa, Pisa, Italy; 4 Diabetes Unit, University Hospital, Pisa, Italy; 5 Unit of Internal Medicine, University Hospital, Pisa, Italy; 6 Department of Medical Sciences, University of Turin, Turin, Italy; 7 Nephrology Unit, “Mons. Dimiccoli” Hospital, Barletta, Italy; 8 Department of Medicine, University of Perugia, Perugia, Italy; 9 Department of Emergency and Organ Transplantation, “Aldo Moro” University, Bari, Italy; 10 Nephrology, Dialysis and Transplantation Unit, “Policlinico” University Hospital, Bari, Italy; 11 Nephrology and Dialysis Unit, Department of Advanced Medical and Surgical Sciences, University of Campania “Luigi Vanvitelli”, Naples, Italy

**Keywords:** Diabetes mellitus, Diabetic nephropathy, Albuminuria, Estimated glomerular filtration rate, End-stage kidney disease, Anti-hyperglycemic therapy

## Abstract

**Aims:**

This joint document of the Italian Diabetes Society and the Italian Society of Nephrology reviews the natural history of diabetic kidney disease (DKD) in the light of the recent epidemiological literature and provides updated recommendations on anti-hyperglycemic treatment with non-insulin agents.

**Data Synthesis:**

Recent epidemiological studies have disclosed a wide heterogeneity of DKD. In addition to the classical albuminuric phenotype, two new albuminuria-independent phenotypes have emerged, i.e., “nonalbuminuric renal impairment” and “progressive renal decline”, suggesting that DKD progression toward end-stage kidney disease (ESKD) may occur through two distinct pathways, albuminuric and nonalbuminuric. Several biomarkers have been associated with decline of estimated glomerular filtration rate (eGFR) independent of albuminuria and other clinical variables, thus possibly improving ESKD prediction. However, the pathogenesis and anatomical correlates of these phenotypes are still unclear. Also the management of hyperglycemia in patients with type 2 diabetes and impaired renal function has profoundly changed during the last two decades. New anti-hyperglycemic drugs, which do not cause hypoglycemia and weight gain and, in some cases, seem to provide cardiorenal protection, have become available for treatment of these individuals. In addition, the lowest eGFR safety thresholds for some of the old agents, particularly metformin and insulin secretagogues, have been reconsidered.

**Conclusions:**

The heterogeneity in the clinical presentation and course of DKD has important implications for the diagnosis, prognosis, and possibly treatment of this complication. The therapeutic options for patients with type 2 diabetes and impaired renal function have substantially increased, thus allowing a better management of these individuals.

## Introduction

Diabetic nephropathy is a major long-term complication affecting approximately 30% of patients with type 1 diabetes (T1D) and 40% of those with type 2 diabetes (T2D) [1]. Nowadays, it represents the leading cause of end-stage kidney disease (ESKD) worldwide, accounting for approximately 40% of new patients requiring renal replacement therapy [2]. Recently, epidemiological surveys have highlighted the unique heterogeneity of the natural history of this complication, thus prompting the use of “diabetic kidney disease” (DKD) to encompass all types of renal injury occurring in diabetic individuals [3]. In particular, in addition to the classical albuminuric phenotype, two new phenotypes have emerged, i.e., “nonalbuminuric renal impairment” and “progressive renal decline”, which suggest that DKD progression toward ESKD in both T1D and T2D may occur through two distinct pathways heralded by a progressive increase in albuminuria and decline in glomerular filtration rate (GFR), respectively [4]. Furthermore, during the last two decades, the management of hyperglycemia in T2D patients with impaired renal function has profoundly changed, as several new anti-hyperglycemic drugs have become available for treatment of these individuals and the lowest GFR safety thresholds for some of the old agents have been reconsidered [5]. This joint document of the Italian Diabetes Society (SID) and the Italian Society of Nephrology (SIN) extensively reviews the natural history of DKD in the light of the recent epidemiological literature, though a systematic review of the literature was not made. In addition, it provides updated recommendations on anti-hyperglycemic treatment with non-insulin agents in patients with T2D and impaired renal function.

## Natural history of DKD

In the traditional, five-stage natural history of diabetic nephropathy, microalbuminuria represents the first abnormality occurring in individuals suffering from this complication. It later progresses to macroalbuminuria, which in turn precedes GFR decline, usually in parallel with development and progression of retinopathy [6]. For this reason, the screening and diagnosis of diabetic nephropathy have been traditionally based on the assessment of albuminuria [7]. Furthermore, albuminuria has long been considered as the main prognostic factor for both progression to ESKD and morbidity and mortality from cardiovascular disease (CVD) [8]. Finally, clinical trials with renoprotective agents, such as the blockers of the renin-angiotensin system (RAS), have generally tested the efficacy of these drugs in halting progression and/or favoring regression of albuminuria from one category to another [9], based on the assumption that targeting albuminuria in diabetic individuals results in better renal and CVD outcomes [10].

This albuminuria-centric model of the natural history of diabetic nephropathy has been questioned by a number of epidemiological observations accumulated during the last decades on the incidence and prevalence of DKD and its main manifestations, i.e., increased albuminuria and reduced GFR, usually estimated using different formulas (eGFR).

These data indicate that the overall burden of DKD has not decreased during this period. Serial cross-sectional analyses of the National Health and Nutrition Examination Survey (NHANES) data from 1988 through 2014 have shown that, among US adults with diabetes, prevalence of DKD has remained stable during this period [11]. In contrast, serial cross-sectional studies conducted in a Japanese diabetic population have demonstrated that prevalence of DKD has increased from 18.5% in 1996 to 25.6% in 2014 [12]. Finally, data from the National Health Interview Survey, the National Hospital Discharge Survey, the US Renal Data System, and the US National Vital Statistics System have indicated that, among the major diabetic complications, ESKD has shown the smallest decline between 1990 and 2010 among US adults with diabetes, likely due to the marked decrease in the incidence of acute myocardial infarction and stroke, which may have favored DKD progression toward its later stages by reducing mortality from CVD [13].

Conversely, impressive diverging changes have been reported in the prevalence of albuminuria and reduced eGFR. The NHANES data have shown that, from 1988 to 2014, the prevalence of albuminuria has declined by 24% [adjusted prevalence ratio 2009–2014 vs 1988–1994, 0.76 (95% confidence interval 0.65–0.89), P< 0.001], that of macroalbuminuria has remained rather stable [0.82 (0.59–1.14), P= 0.22], and that of eGFR < 60 ml/min/1.73 m^2^ and especially < 30 ml/min/1.73 m^2^ has dramatically increased [1.61 (1.33–1.95), P< 0.001, and 2.86 (1.38–5.9), P< 0.004, respectively] [11]. Similar secular trends in the prevalence of albuminuria and reduced eGFR have been reported in the Japanese diabetic population from 1996 through 2014 [12].

These opposite temporal trends in the prevalence of albuminuria and reduced eGFR reflect the fact that remission/regression of microalbuminuria (and even macroalbuminuria) to normoalbuminuria is an increasingly common feature that far outweighs progression to proteinuria in both T1D [14–16] and T2D [17–19], whereas eGFR loss, once initiated, continue to progress inevitably to ESKD, albeit at widely variable rates. The increasing divergence between albuminuria and reduced eGFR challenges the classical view that albuminuria invariably precedes and sustains eGFR loss, suggesting that both initiation and progression of renal function decline may occur also independently of the development of albuminuria and its subsequent course. This concept is supported by the emergence of two new phenotypes, i.e., nonalbuminuric renal impairment and progressive renal decline.

Box 1 During the last decades, prevalence of DKD has not decreased and incidence of ESKD has decreased only slightly, with major changes in the two main DKD manifestations, i.e., albuminuria, the prevalence of which has decreased (with macroalbuminuria remaining stable), and reduced eGFR, the prevalence of which has increased (especially for eGFR < 30 ml/min/1.73 m^2^).

### Nonalbuminuric renal impairment and progressive renal decline

Two early studies reported that reduction of creatinine clearance may occur in patients with both T1D and T2D who remain normoalbuminuric [20, 21]. These observations have been confirmed in the last decades, during which the prevalence of the nonalbuminiric phenotype has increased among individuals with T2D (Table 1) and, though to a lower extent, also with T1D (Table 2).

**Table 1:** Distribution of DKD phenotypes in individuals with T2D

Study	Country	Years	GFR method	*N*	T2D, %	Alb^−^ eGFR^−^, %	Alb^+^ eGFR^−^, %	eGFR^+^, %				
								All, %	Alb^+^, %	Alb, %	Alb^−^, % of all eGFR^+^	Alb^−^/Ret^−^, % of all eGFR^+^
*Cross-sectional serial (NHANES)*												
Kramer et al. [22] (NHANES 1988–1994)	US	1988–1994	MDRD	1197	100	54.0	31.7	14.3	9.3	5.0	35.1	29.8
Afkarian et al. [23] (NHANES 1988–1994)	US	1988–1994	CKD-EPI	1430	100	54.0	27.1	19.0	10.3	8.7	45.8	–
Bailey et al. [24] (NHANES 1999–2012)	US	1999–2012	CKD-EPI (+ MDRD)	2915	100	51.0	24.0	25.0	13.1	11.9	47.7	–
				1466 ≥ 65 years	100	38.7	20.4	40.8	21.3	19.5	47.7	–
Mottl et al. [25] (NHANES 2001–2008)	US	2001–2008	CKD-EPI	2798	100	56.5	23.0	20.5	9.9	10.6	51.8	–
*Cross-sectional (observational)*												
MacIsaac et al. [26]	Australia	1990–2001	Isotopic	301	100	38.2	25.6	36.3	21.9	14.3	39.4	29.3
Dwyer et al. [27] (DEMAND Global)	33 countries from Europe, Asia, Africa, Oceania, North & Central-South America	2003	MDRD	11,573	100	43	34	23	13	9	40.1	–
Yokoyama et al. [29] (JDDM)	Japan	2004–2005	MDRD Jap	3297	100	61.8	22.9	15.3	7.4	7.9	51.8	39.9
Thomas et al. [30, 31] (NEFRON)	Australia	2005	MDRD	3893	100	52.9	24.2	22.9	10.5	12.4	54.1	–
Penno et al. J Hypertens. 2011;29:1802–1809 (RIACE)	Italy	2006–2008	MDRD (+ CDD-EPI)	15,773	100	62.5	18.7	18.8	8.2	10.6	56.6	43.2
Afghahi et al. J Diabetes Complications. 2013;27: 229–234 (Swedish National Diabetes Register)	Sweden	2007	MDRD (+ Cockcroft-Gault)	81,315	100	63.5	16.4	20.0	7.6	12.4	61.9	49.6
Hill et al. [34] (UK National Diabetes Audit)	UK	2007–2008	CKD-EPI	800,439	100	57.7	17.7	24.5	8.9	15.6	63.7	–
Koye et al. [40] (CRIC)	US	2003–2008	CKD-EPI	1908^a^ (eGFR 21–44 years > 20 < 70, 45–64 years < 60, 65–74 years < 50)	100	–	–	100.0	71.6	28.4	28.4	
De Cosmo et al. [35] (AMD Annals)	Italy	2009	CKD-EPI	120,903	100	52.6	23.8	23.5	12.2	11.3	48.2	39.3
Gao et al. [36]	China	2008–2009	CKD-EPI	8811	100	67.2	17.5	15.3	4.6	10.7	69.9	–
Lee et al. [39]	Korea	2011–2013	MDRD (+ CKD-EPI)	1067^a^	100	30.1	31.1	38.8	29.6	9.2	23.7	17.1
Rodriguez-Poncelas et al. [37] (PERCEDIME2)	Spain	2011	MDRD	1145	100	62.1	9.9	18.0	12.5	5.5	69.4	–
Bramlage et al. [38] (DPV/DIVE Registry)	Germany	2010–2017	MDRD	240,510	100	45.1	15.8	39.3	12.4	26.9	68.3	–
*Cross-sectional (intervention)*												
Drury et al. [41] (FIELD)	Australia, New Zealand, Finland	1998–2000	MDRD	9795	100	71.2	23.4	5.3	2.2	3.1	59.1	–
Ninomiya et al. [42] (ADVANCE)	20 countries from Europe, Asia, Oceania, North America	2001–2003	MDRD	10,640	100	57.5	23.3	19.2	7.3	11.8	61.6	–
Tobe et al. [43] (ONTARGET &TRASCEND)	40 countries from Europe, Asia, Oceania, North America	2001–2004	MDRD	23,422	37.5	61.9	14.1	24.0	7.6	16.4	68.2	–
Bakris et al. [44] (ACCOMPLISH)	US, Denmark, Sweden, Norway, Finland	2003–2005	?	8519	60	62.3	27.2	10.5	5.6	4.9	46.8	–
*Longitudinal*												
Retnakaran et al. [49] (UKPDS)	UK	1977–1991	Cockcroft-Gault	4006 (median follow-up of 15 years)	100	47.3	24.4	28.3	13.9 (9.3)^b^	14.4 (19.0)^b^	50.8 (67.1)^b^	–

^a^Exclusion of patients on RAS blockers

^b^% values at the time reduction of eGFR occurred

*Alb*^*+*^ micro or macroalbuminuria, *Alb*^*−*^ normoalbuminuria, *eGFR*^+^ < 60 ml/min/1.73 m^2^, *eGFR*^−^ ≥ 60 ml/min/1.73 m^2^, *Ret*^−^ no retinopathy, *NHANES* National Health And Nutrition Examination Survey, *DEMAND* Developing Education on Microalbuminuria for Awareness of Renal and Cardiovascular Risk in Diabetes, *JDDM* Japan Diabetes clinical Data Management, *NEFRON* National Evaluation of the Frequency of Renal Impairment cO-existing with NIDDM, *RIACE* Renal Insufficiency And Cardiovascular Events, *CRIC* Chronic Renal Insufficiency Cohort, *PERCEDIME2* Prevalence of ease in Patients with Type 2 Diabetes, *DPV* Diabetes-Patienten-Verlaufsdokumentation, *DIVE* DIabetes Versorgungs-Evaluation, *FIELD* Fenofibrate Intervention and Event Lowering in Diabetes, *ADVANCE* Action in Diabetes and Vascular disease: preterAx and diamicroN-MR Controlled Evaluation, *ONTARGET*/*TRASCEND* Ongoing Telmisartan Alone and in Combination With Ramipril Global End Point Trial/Telmisartan Randomized Assessment Study in ACE Intolerant Subjects With Cardiovascular Disease, *ACCOMPLISH* Avoiding Cardiovascular events through COMbination therapy in Patients LIving with Systolic Hypertension, *UKPDS* United Kingdom Prospective Diabetes Study

**Table 2:** Distribution of DKD phenotypes in individuals with T1D

Study	Country	Years	GFR method	*N*	T1D, %	Alb^−^ eGFR^−^, %	Alb^+^ eGFR^−^, %	eGFR^+^, %				
								All, %	Alb^+^, %	Alb, %	Alb^−^, % of all eGFR^+^	Alb^−^/Ret^−^, % of all eGFR^+^
*Cross-sectional*												
Thorn et al. [45] (FinnDiane)	Finland	1998–2005	CKD-EPI	3809	100	67.4	19.4	13.1	11.1	2.0	15.5	–
Penno et al. [32]	Italy	2001–2009	MDRD	777	100	89.4	6.8	3.7	1.5	2.2	58.6	11.1
Pacilli et al. [47] (AMD-Annals)	Italy	2004–2011	CKD-EPI	20,464	100	76.5	15.4	8.0	4.1	3.9	48.9	–
Lamacchia et al. [48] (AMD-Annals)	Italy	2004–2011	CKD-EPI	1395 (eGFR ≤ 60 ml/min/1.73 m^2^)	100	–	–	100.0	48.5	51.5	51.5	36.6
Hill et al. [34] (UK National Diabetes Audit)	UK	2007–2008	CKD-EPI	68,177	100	67.6	18.4	14.0	6.4	7.6	54.4	–
*Longitudinal*												
Molitch et al. [50] (DCCT/EDIC)	North America	1983–1989	MDRD	1439 (mean follow-up of 19 years)	100	46.9	46.9	6.2	4.7	1.5	23.6	–

*Alb*^+^ micro or macroalbuminuria, *Alb*^−^ normoalbuminuria, *eGFR*^+^ < 60 ml/min/1.73 m^2^, *eGFR*^−^ ≥ 60 ml/min/1.73 m^2^, *Ret*^−^ no retinopathy, *FinnDiane* Finnish Diabetic Nephropathy Study, *DCCT*/*EDIC* Diabetes Control and Complications Trial/Epidemiology of Diabetes Interventions and Complications

A cross-sectional analysis of US adults with diabetes from the NHANES 1988–1994 showed that 35.1% of subjects with an eGFR < 60 ml/min/1.73 m^2^, as calculated using the Modification of Diet in Renal Disease (MDRD) formula, were normoalbuminuric, and that albuminuria and retinopathy were both absent in 29.8% of patients with reduced eGFR [22]. Subsequent cross-sectional analyses of the NHANES data showed higher adjusted prevalence rates (~ 50%) for the nonalbuminuric phenotype among individuals with reduced eGFR, as calculated using the Chronic Kidney Disease Epidemiology Collaboration (CKD-EPI) equation, i.e., 45.8%, in the years 1988–1994 [23], 47.7%, in the years 1999–2012 [24], 51.8%, in the years 2001–2008 [25], and 48.1%, in the years 2005–2008 [11]. These data are consistent with the decreasing prevalence of albuminuria and the increasing prevalence of reduced eGFR reported among US [11] and Japanese [12] adults with diabetes.

Similar findings have emerged from cross-sectional studies in cohorts of T2D patients from several countries. McIsaac et al. reported that, among 301 patients with T2D attending an outpatient clinic in Australia in the years 1990–2001, 39.4% of those with an GFR < 60 ml/min/1.73 m^2^, as measured by an isotopic method, were normoalbuminuric [26]. All the surveys conducted in the subsequent years reported a rising prevalence (increasing approximately from 40 to 70%) of the nonalbuminuric phenotype among T2D patients with reduced eGFR, with differences among studies depending also on the geographic area and the formula used for eGFR calculation. In detail, prevalence was: 40.1% in the Developing Education on Microalbuminuria for Awareness of renal and cardiovascular risk in Diabetes (DEMAND) Study (multinational, MDRD, 2003) [27, 28]; 51.8% in the Japan Diabetes Clinical Data Management (JDDM) Study (Japan, MDRD, 2004–2005) [29]; 54.2% in the National Evaluation of the Frequency of Renal Impairment cO-existing with NIDDM (NEFRON) (Australia, MDRD, 2005) [30, 31]; 56.6% in the Renal Insufficiency And Cardiovascular Events (RIACE) Italian Multicenter Study (Italy, MDRD, 2006–2008) [32]; 61.9% in an analysis of the Swedish National Diabetes Register (Sweden, MDRD, 2007) [33]; 63.7% in the UK National Diabetes Audit (UK, CKD-EPI, 2007–2008) [34]; 48.2% in the AMD-Annals Initiative (Italy, CKD-EPI, 2009) [35]; 69.9% in a Chinese cohort (China, CKD-EPI, 2008–2009) [36]; 69.4% in the Prevalence of ease in Patients with Type 2 Diabetes (PERCEDIME2) Study (Spain, MDRD, 2011) [37] and 68.3% in the Diabetes-Patienten-Verlaufsdokumentation (DPV) and DIabetes Versorgungs-Evaluation (DIVE) registries (Germany, MDRD, 2010–2017) [38]. Lower prevalence rates were reported in two epidemiological surveys from Korea (23.7%) [39] and US [the Chronic Renal Insufficiency Cohort (CRIC) Study, 28.4%] [40], but patients whose albuminuria status was possibly related to RAS blocker therapy were excluded from these analyses.

A high prevalence of the nonalbuminuric phenotype (ranging approximately from 45 to 70%) was also detected in T2D patients enrolled in multicenter multinational interventional studies, in which however values were affected by the different entry criteria. In detail, prevalence was: 59.1% in the Fenofibrate Intervention and Event Lowering in Diabetes (FIELD) Study (MDRD, 1998–2000) [41]; 61.6% in the Action in Diabetes and Vascular disease: preterAx and diamicroN-MR Controlled Evaluation (ADVANCE) Study (MDRD, 2001–2003) [42]; 68.2% in the Ongoing Telmisartan Alone and in Combination with Ramipril Global Endpoint Trial (ONTARGET) and Telmisartan Randomised AssessmeNt Study in ACE iNtolerant subjects with cardiovascular Disease (TRASCEND) Study (MDRD, 2001–2004) [43]; and 46.8% in the Avoiding Cardiovascular Events in Combination Therapy in Patients Living with Systolic Hypertension (ACCOMPLISH) Study (MDRD, 2003–2005) [44].

Altogether, these data support the concept that prevalence of nonalbuminuric renal impairment in T2D has increased during the last decades and that it has now become the prevailing phenotype among patients with reduced eGFR. Currently, it can be estimated that, among T2D individuals, 50–65% have no DKD, 20–30% have albuminuria alone (i.e., albuminuric DKD with preserved eGFR), and 15–25% have reduced eGFR, the majority of them (8–16%) with normoalbuminuria (i.e., nonalbuminuric DKD or reduced eGFR alone) and the remaining with micro or macroalbuminuria (i.e., albuminuric DKD with reduced eGFR or combination of albuminuria and reduced eGFR) (Table 1).

A high prevalence of the nonalbuminuric phenotype has been observed also in individuals with T1D. A cross-sectional analysis of the Finnish Diabetic Nephropathy (FinnDiane) Study cohort detected that 15.5% of the 502 T1D patients with reduced eGFR were normoalbuminuric (Finland, CKD-EPI, 1998–2005) [45]. However, more recent cross-sectional studies from Italy and UK reported a much higher prevalence (approximately 50–60%) of the nonalbuminuric phenotype among T1D patients with reduced eGFR, i.e., 58.6% in a cohort study from Tuscany (Italy, MDRD, 2001–2009) [46]; 48.9% and 51.5% in the AMD-Annals Initiative (Italy, CKD-EPI, 2004–2011) [47, 48]; and 54.4% in the UK National Diabetes Audit (UK, CKD-EPI, 2007–2008) [34]. These data seem to indicate that prevalence of nonalbuminuric renal impairment is increasing also in T1D and that, nowadays, it is at least as frequent as the albuminuric phenotype among T1D individuals with impaired renal function.

Longitudinal analyses of patients with T2D from the United Kingdom Prospective Diabetes Study (UKPDS) and of patients with T1D from the Diabetes Control and Complications Trial (DCCT)/Epidemiology of Diabetes Interventions and Complications (EDIC) provided information on the course of albuminuria and reduced eGFR in these individuals. In the UKPDS, of the 1132 individuals (28.3% of the overall cohort) who developed reduced eGFR over a 15-year follow-up, 67.1% were normoalbuminuric and 50.8% remained in this category, whereas 16.3% became microalbuminuric thereafter (UK, Crockcroft-Gault) [49]. Likewise, in the DCCT/EDIC Study, of the 89 individuals (6.2% of the overall cohort) developing reduced eGFR during a 19-year follow-up, 23.6% were normoalbuminuric (North America, MDRD) [50]. These data indicate not only that eGFR may decline prior to the increase of albuminuria, but also that reduced eGFR may remain the sole renal abnormality in a substantial proportion of patients with DKD or become associated with albuminuria only later. Thus, albuminuric DKD with reduced eGFR represents a heterogeneous DKD phenotype, including individuals progressing along the classical pathway characterized by eGFR decline only after the development and progression of microalbuminuria and those presenting initially with nonalbuminuric renal impairment and developing albuminuria only at a later stage.

Finally, Krolewski et al. identified the phenotype of progressive renal decline by analyzing the slope of eGFR in diabetic patients enrolled in the Joslin Kidney Studies [51]. This phenotype was observed in 19% of T1D and 28% of T2D individuals [52] and now accounts for the majority of ESKD cases in T1D [53]. It is characterized by eGFR loss which occurs early (or late) in the natural history of diabetic nephropathy, while patients have normal renal function, and progresses unidirectionally to ESKD at a variable rate, from slow to very fast [52]. Progression was shown to be mainly linear, with only a small percentage of patients exhibiting non-linear decline with acceleration or deceleration [53, 54], though the use of a modeling method designed to handle heterogeneity revealed that non-linear trajectories are indeed common in patients with T2D [55]. It can be diagnosed using serial measurement of serum creatinine and/or cystatin C, which allow to estimate the slope of eGFR when it is still within the normal range [51, 52]; decliners are usually identified by an eGFR loss ≥ 3 ml/min/year, whereas a rapid progression is defined as an eGFR loss ≥ 5 ml/min/year according to the Kidney Disease: Improving Global Outcomes (KDIGO) guidelines [56]. Of note, both initiation and progression of eGFR decline may be independent of albuminuria. In fact, progressive renal decline was observed among patients with any level of albuminuria, though it was less frequent among individuals with normoalbuminuria (9% in T1D and 20% in T2D) than in those with microalbuminuria (22% in TID and 33% in T2D) and macroalbuminuria (51% in TID and 68% in T2D) [51, 52, 57–59]. Conversely, most of the normoalbuminuric individuals maintained stable renal function over time [52, 58], but a substantial proportion of non-decliners was observed also among proteinuric patients [52, 59]. In addition, in both decliners and non-decliners, albuminuria may either progress, remain stable, or regress, though progression is more frequent among decliners and regression is more frequent among non-decliners. Indeed, in a cohort of 79 microalbuminuric patients with T1D, microalbuminuria progressed to macroalbuminuria in 12 (50.0%) of the 24 decliners and in 10 (22.2%) of the 45 non-decliners, whereas it regressed in 3 decliners (12.5%) and 24 non-decliners (53.3%) [51].

**Fig. 1:**
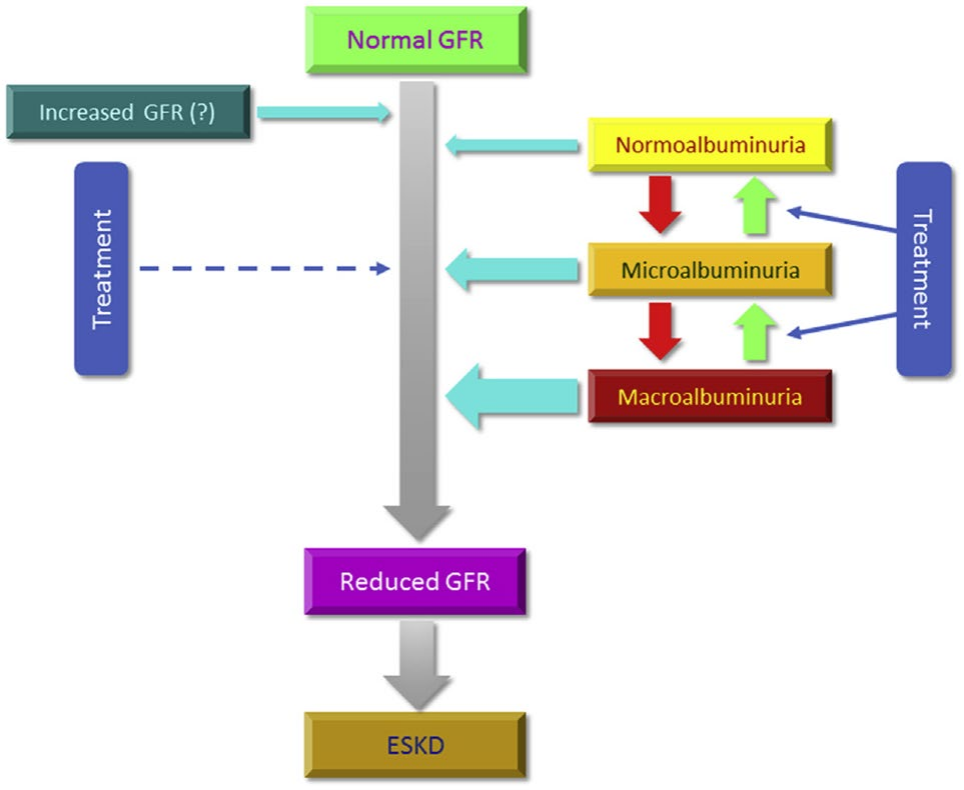
Albuminuric and nonalbuminuric pathways of DKD progression. *DKD* diabetic kidney disease, *GFR* glomerular filtration rate, *ESKD* end-stage kidney disease

Taken together, these findings indicate that albuminuria and reduced eGFR may occur and proceed either together or separately as complementary or “twin” manifestations of DKD [60] and that there are two main pathways for the onset and progression of DKD, i.e., albuminuric and nonalbuminuric (Fig. 1). In the classical albuminuric pathway, eGFR loss is preceded and substantially driven by the development and progression of microalbuminuria, the reduction of which is therefore expected to significantly slow down renal function decline. In the emerging nonalbuminuric pathway, of which nonalbuminuric renal impairment and progressive renal decline are two sides of the same coin, eGFR loss is independent from ← of microalbuminuria and, hence, it may not benefit from reduction of albuminuria. As such, it either occurs in the absence of albuminuria (or right before or soon after the onset of microalbuminuria) or progresses toward ESKD irrespective of whether albuminuria remains stable, progresses or reverses.

However, the level of albuminuria [61], from increments within the normal range [62] to nephrotic range proteinuria [63], remains a powerful independent predictor of eGFR decline, especially in diabetic individuals with low eGFR. A recent observational study evaluated cardiorenal risk in diabetic (n = 693) versus non-diabetic (n = 1491) patients with chronic kidney disease (CKD) (75% with an eGFR < 45 ml/min/1.73 m^2^), stratified by the level of proteinuria and followed for a median of 4.07 years [64]. In the absence of proteinuria (< 0.15 g/24 h), diabetic patients were not exposed to an increased risk of ESKD compared with non-diabetic individuals, whereas they had only a higher CVD risk in the presence of moderate proteinuria (0.15–0.49 g/24 h). In contrast, in patients with proteinuria ≥ 0.50 g/24 h, the cardiorenal risk was primarily driven by the level of proteinuria independent of the diabetic status [64]. Similar data have been provided by the CRIC Study in the US that prospectively followed 1908 patients with T1D or T2D and reduced eGFR (mean eGFR 41 ml/min/1.73 m^2^) for a median of 6.3 years [40].

Complexity of this issue further increases when considering that, in the context of low eGFR, the absolute level of proteinuria does have an intrinsic pathophysiological limitation, as it depends not only on the extent of kidney damage but also on the number and function of residual nephrons; therefore, a low proteinuria level can be merely a consequence of low eGFR. In this regard, a recent multi-cohort prospective study in 3957 patients (29% with diabetes) with an eGFR < 60 ml/min/1.73 m^2^ has demonstrated that proteinuria indexed to eGFR acts as an independent predictor of ESKD, with this association being stronger than that observed with absolute proteinuria level and in diabetic than in non-diabetic individuals [65].

Given the strong association between albuminuria and eGFR decline, several studies have investigated whether reduction of albuminuria translates into improved renal outcomes in the long-term. A pooled analysis of interventional studies showed that, in both types of diabetes, the initial decrease in albuminuria with anti-hypertensive treatment does not predict the subsequent decline in eGFR in early nephropathy (microalbuminuria and preserved eGFR), but it does in advanced disease (macroalbuminuria and reduced eGFR) [66]. In fact, in the DCCT/EDIC Study, remission of microalbuminuria in T1D patients was not associated with a significant reduction in the risk of adverse outcomes, including sustained eGFR < 60 ml/min/1.73 m^2^ [67], whereas in the ADVANCE Study, a “real” decrease in albuminuria in T2D individuals was associated with a significantly lower risk of a composite primary cardiorenal outcome, but not of major renal events [68]. Conversely, a post hoc analysis of the Reduction of Endpoints in NIDDM with the Angiotensin II Antagonist Losartan (RENAAL) Study clearly showed that not only baseline proteinuria, but also changes in proteinuria in the first 6 months of therapy were related to the degree of long-term renal protection in proteinuric patients with T2D [69]. Recently, an observational study from the Stockholm CREAtinine Measurements (SCREAM) project [70] and two patient-level meta-analyses [71, 72] including as many as 31,732, 29,979, and 693,816 CKD patients (61%, 71%, and 80% with diabetes), respectively, provided conclusive evidence that a decrease in albuminuria is associated with a reduction of the subsequent risk of ESKD, depending on the level of albuminuria [71, 72]. Collectively, current evidence supports the use of changes in albuminuria as a surrogate outcome in trials designed to test the efficacy of interventions aimed at halting the progression of DKD, in the setting of increased albuminuria [73].

Despite the large body of evidence indicating the existence of different DKD phenotypes, it is still unclear whether the albuminuric and nonalbuminuric DKD models represent true distinct pathways underlying different pathogenic and pathophysiological mechanisms and what is the reason for the progressive switch from the classical albuminuric presentation to the new nonalbuminuric phenotypes, i.e., nonalbuminuric renal impairment and progressive renal decline.

Box 1.1 In the last decades, two new phenotypes have been increasingly recognized: “nonalbuminuric renal impairment”, in which eGFR decline is not preceded by the development and progression of microalbuminuria and may remain the sole renal abnormality, and “progressive renal decline,” in which eGFR loss represents the main abnormality that develops and progresses independently of the presence and extent of albuminuria and its subsequent course. These phenotypes suggest that DKD onset and progression may occur also through a “nonalbuminuric” pathway, distinct from the classical “albuminuric” pathway. However, when present, albuminuria remains a strong predictor of eGFR decline and a main target of renoprotective therapy, especially in the setting of moderate-to-severe impairment of renal function.

### Impact of improved treatment on the natural history of diabetic nephropathy

The opposite temporal trends in the prevalence of albuminuria and reduced eGFR and the increasingly divergent presentation and course of these two main DKD manifestations observed over the last decades suggest that changes in the natural history of diabetic nephropathy may be related to changes in the type and intensity of preventive and therapeutic interventions aimed at controlling the known risk factors for the development and progression of diabetic complications, including DKD. Indeed, serial cross-sectional analyses of data from US and Japanese adults with diabetes have shown an increasing use of glucose-lowering medications, RAS blockers, and statins, which has resulted in a progressive improvement in glycemic, blood pressure and lipid control from the 90s to the 10s [11, 12] suggesting a cause–effect relationship with the reduction in the prevalence of albuminuria and the increment in the prevalence of reduced eGFR. However, while the relation with reduction of albuminuria is well established, it is difficult to understand whether and how changes in treatment resulted in an increment of impaired eGFR.

One possible explanation is the progressive decrease in all-cause and CVD mortality observed in diabetic individuals as a result of improved treatment [74], which may have favored progression toward impaired eGFR. In addition, the increasing age of the population due to the reduced mortality may have resulted in an increased prevalence of reduced eGFR. However, data from the NHANES argue against this hypothesis, as the increase in prevalence of reduced eGFR was observed both in younger and older individuals [11] and reduction in mortality was confined to individuals with albuminuria [75]. Rather, the monotonic increase in diabetes duration with no change in mean age reported in the NHANES cohort from 1988 to 2014 [11] suggests a progressively earlier onset of T2D, which was found to be an independent predictor of eGFR decline [76]. Another explanation is the progressive lowering of average blood pressure during the past two decades among adults with diabetes [11, 12], which may have resulted in reduction of renal perfusion pressure and, hence, of eGFR in some individuals. Finally, the opposite temporal trends in the prevalence of albuminuria and reduced eGFR have been related to the use RAS blockers. These agents, in addition to favoring the prevention and/or regression of micro/macroalbuminuria to normoalbuminuria [9], cause a reversible, hemodynamically-mediated eGFR drop that may be of clinical significance [77], though in the long run they slow down eGFR decline [78], possibly through their anti-proteinuric effect [69]. This interpretation is supported by the finding that, during the last decades, use of RAS blockers and prevalence of the nonalbuminuric phenotype have increased in parallel. For instance, in the NHANES, use of these agents (weighed % and 95% confidence interval) increased from 24.4% (21.0–28.3%) in 1998–1994 to 56.2% (52.3–59.9%) in 2009–2014 [11], whereas recent surveys reported values up to 70% or more [32, 33, 35, 45]. The much lower prevalence of nonalbuminuric renal impairment when patients on RAS blockers were excluded from the analysis [39, 40] is also consistent with the concept that individuals with the nonalbuminuric phenotype are those who either did not develop albuminuria or were microalbuminuric at some point of the natural history of DKD but later became normoalbuminuric because of treatment with RAS blockers, i.e., in the absence of anti-RAS treatment, these patients would have presented with the classical albuminuric phenotype.

However, several lines of evidence argue against this interpretation and support the existence of two distinct pathways, albuminuric and nonalbuminuric, to DKD progression. First, a relation between use of RAS blockers and remission/regression of albuminuria has emerged in some studies [17, 18], but not in others [14, 19, 57, 58, 79]. In addition, in both cross-sectional and longitudinal studies, the use of RAS blockers was not higher (and in some cases it was even lower) in T1D and T2D individuals with nonalbuminuric DKD as compared with those with albuminuria and either preserved or reduced eGFR [31, 32, 35, 45, 50] and a substantial proportion of patients with the nonalbuminuric phenotype was not on these agents [31–33, 35, 49, 50]. These data indicate that albuminuric DKD may develop despite RAS blocker treatment and that the nonalbuminuric phenotype may occur independently of such therapy. Second, the opposite trends in the absolute prevalence of albuminuria and impaired eGFR are in contrast with the finding that reducing albuminuria with RAS blockers decreases eGFR loss in individuals with both T1D and T2D, especially in those with proteinuria [80–82]. Third, previous studies in T2D patients have shown that the independent correlates of reduced eGFR and albuminuria differ between each other, i.e., female gender, non-smoking status, age, and diabetes duration for reduced eGFR and male gender, former or current smoking status, hemoglobin (Hb) A_1c_, body mass index, waist circumference, and retinopathy for albuminuria [29, 49, 83]. Fourth, nonalbuminuric renal impairment was found to be associated with distinct features which recapitulate the correlates of reduced eGFR. Studies in T2D patients have in fact shown that, compared with individuals with the albuminuric forms, those presenting with the nonalbuminuric phenotype were more frequently female, non-smoker, older, and with longer disease duration, though differences in age and years spent with diabetes were observed only versus individuals with albuminuric DKD with preserved eGFR [31, 32, 35]. In addition, at variance with the albuminuric forms, the nonalbuminuric phenotype showed no or weak association with HbA_1c_ and HbA_1c_ variability, hypertension, and the other major microvascular complication of diabetes, i.e., retinopathy, with up to approximately 30–50% of individuals with reduced eGFR showing neither albuminuria nor retinopathy [22, 29, 32, 33, 35]. Studies in T1D patients have found an association with age, but also with HbA_1c_, whereas no relation was detected with smoking status [57, 58].

Altogether, these findings support the concept that changes in treatment, including but not limited to the use of RAS blockers, have unraveled the existence of the two pathways by differentially affecting albuminuria and reduced eGFR. By decreasing albuminuria, improved treatment has been effective in reducing DKD progression through the classical albuminuric pathway. Conversely, due to the insufficient effect of these agents on eGFR decline, improved treatment has failed to reduce DKD progression through the nonalbuminuric pathway but, by favoring prevention and/or regression of albuminuria, it has unmasked the new phenotypes, nonalbuminuric renal impairment and progressive renal decline.

In parallel with the increasing recognition of the nonalbuminuric pathway and the new albuminuria-independent DKD phenotypes, several studies have been performed to identify novel biomarkers of eGFR decline which might shed light on the pathogenic mechanisms underlying the nonalbuminuric pathway and improve prediction of DKD progression independent of albuminuria.

Box 1.2 Improvements in diabetes management over the last decades, with increasing use of medications, especially RAS blockers, resulting in better glycemic, blood pressure and lipid control, have been effective in reducing the prevalence of albuminuria, but not that of low eGFR. The increased prevention and/or regression of albuminuria due to improved treatment has unmasked the new phenotypes, nonalbuminuric renal impairment and progressive renal decline, indicating the existence of a nonalbuminuric pathway of DKD onset and progression which is independent of albuminuria.

### Biomarkers of eGFR decline beyond albuminuria

In the recent years, a number of studies in both T1D and T2D patients have identified several serum and urine biomarkers that correlate with eGFR decline beyond albuminuria and other clinical variables and improve prediction of ESKD.

An independent association between serum uric acid levels in the high-normal range and eGFR decline was detected in patients with both T1D [84, 85] and T2D [86–92] and also in non-diabetic individuals [93]. The association in patients with T2D was confirmed by a recent meta-analysis [92] and appeared to be restricted to individuals with preserved renal function at baseline [94]. How serum uric acid can incite eGFR loss is not completely understood, but pro-inflammatory mechanisms have been suggested [95]. Based on these findings, serum uric acid has been proposed as a target for treatment of CKD [96] and a trial with allopurinol is currently on-going in patients with T1D [97].

Among inflammatory markers, circulating levels of tumor necrosis factor (TNF) receptors 1 and 2, but not of free and total TNFα, were consistently found to be associated with eGFR decline in patients with either T1D [98–100] or T2D [91, 101–105] and to improve prediction of ESKD when added to algorithms including clinical variables [91, 102]. Other markers of inflammation that were found to be independently associated with eGFR decline include circulating interleukin (IL)-6 [106] and C-reactive protein [107] and urinary monocyte chemotactic protein-1 (MCP-1) [108], in patients with T2D, and multiple urinary inflammatory markers (IL-6, IL-8, MCP-1, interferon-γ-inducible protein, and macrophage inflammatory protein-1δ) in patients with T1D [109].

Markers of tubular injury have also been associated with eGFR decline in both types of diabetes. In detail, the following markers were found to be independent predictors of eGFR loss: serum kidney injury molecule-1 (KIM-1), in patients with T1D [110], and urinary levels of KIM-1 [111–113], β2-microglobulin [113], liver-type fatty acid-binding protein (FABP) [114], and nonalbumin protein [115], and serum retinol-binding protein 4 (RBP4) [107], in patients with T2D. However, other studies failed to demonstrate an independent association of markers of tubular injury with eGFR decline [116, 117].

Other biomarkers that have been associated with eGFR loss include: urinary high molecular weight adiponectin [118], adiponectin [119], type IV collagen [120, 121], and haptoglobin [122, 123]; circulating arginine vasopressin, as measured as copeptin [124], adipocyte FABP [125], fibroblast growth factor 21 [126], kininogen and kininogen fragments [127], the angiogenic factor leucine-rich a-2 glycoprotein 1 [128], the anti-ageing hormone soluble Klotho (low levels) [129], and leptin (both high and low levels) [130]; and erythrocyte total polyunsaturated fatty acids (PUFAs), n-3 PUFAs, and n-3/n-6 PUFA ratio, but not n-6 PUFAs (low levels) [106], all in T2D patients (except urinary collagen IV, in both T1D and T2D individuals). In addition, CKD273, a multidimensional urinary proteome classifier consisting of 273 protein fragments, predicted deterioration of renal function in patients with [131] and without [132] albuminuria and also development of microalbuminuria in normoalbuminuric individuals [133]. Finally, panels of multiple markers representing different disease pathways and including inflammatory and tubular biomarkers, were shown to improve prediction of eGFR decline in patients with T2D beyond traditional risk factors [134–139].

An association with eGFR decline was described also for CVD biomarkers, especially high-sensitivity troponin T [140] and left ventricular ejection fraction [141], possibly reflecting the contribution of chronic cardiac dysfunction to progressive eGFR impairment in the context of type 2 cardio-renal syndrome [142]. In addition, arterial stiffness, a marker of arteriosclerosis, was found to be negatively associated with eGFR [143] and to independently predict eGFR decline [141, 144], possibly reflecting the contribution of highly pulsatile pressure and flow to small vessel disease in the kidney [145]. Moreover, renal function decline in T2D individuals was found to be associated with multiple modifiable CVD risk factors [146] and with presence of non-alcoholic fatty liver disease [147].

Finally, hyperfiltration, which has been hypothesized to predispose to irreversible nephron damage [148], was also found to be associated with eGFR decline in both T1D [149] and T2D [150] patients, thus suggesting that it may serve as a predictor of eGFR loss.

These findings indicate that eGFR decline is associated with multiple pathways which may specifically impact on renal function independent of albuminuria and drive DKD onset and progression through the nonalbuminuric pathway.

Box 1.3 Several biomarkers, including uric acid, markers of inflammation, especially TNF receptors 1 and 2, and markers of tubular injury have been shown to be associated with eGFR decline independent of albuminuria and other clinical variables. Other independent correlates of eGFR loss include markers of CVD and arteriosclerosis. An association with hyperfiltration has also emerged.

### Pathogenic mechanisms and anatomical correlates of eGFR decline independent of albuminuria

The clinical and biochemical features associated with nonalbuminuric renal impairment and progressive renal decline support the concept that the pathogenesis of these phenotypes differs from that of the albuminuric ones and suggest the involvement of mechanisms operating mainly at the vascular and/or tubulo-interstitial level.

The hypothesis of a predominant (macro)vascular nature of lesions underlying these phenotypes is supported by the weak or no association of nonalbuminuric renal impairment with diabetic retinopathy and HbA_1c_ [22, 32, 151] and by the relationship of eGFR decline with CVD biomarkers and arterial stiffness, suggesting the involvement of intrarenal arteries. This is more likely in individuals with T2D, who present with several CVD risk factors in addition to hyperglycemia, including hypertension, dyslipidemia, central obesity, and aging itself, all of which may contribute to renal injury, though to a varying extent in each individual [4, 152].

The hypothesis of a predominant tubulo-interstitial nature of lesions underlying these phenotypes is supported by the association of eGFR decline with uric acid [84–94] and markers of inflammation [91, 98–109] and tubular injury [110–115]. In addition, two small studies in patients with biopsy-proven diabetic nephropathy showed that the score for interstitial fibrosis and tubular atrophy was an independent predictor of eGFR decline [153, 154]. It has been suggested that unresolved and/or repeated episodes of acute kidney injury (AKI) may contribute to eGFR decline in diabetic individuals [4], consistent with the demonstration that AKI is a risk factor for future development (or progression) of CKD, depending on its severity, duration, and frequency [155]. Though this hypothesis is unlikely in T1D patients, because eGFR trajectories were shown to be mostly linear in these individuals [53], it cannot be excluded in patients with T2D [156], who are more susceptible to AKI because of the presence of several additional risk factors, such as preexisting CKD, advanced age, heart failure, and hypertension [155, 157].

Unfortunately, there are no or insufficient renal biopsy data to confirm the hypothesis of prevailing (macro)vascular and/or tubulo-interstitial lesions underlying the nonalbuminuric pathway, as compared to the typical microvascular lesions with predominant glomerular injury (glomerular basement membrane thickening, mesangial expansion, and nodular or diffuse glomerulosclerosis) characterizing the classical albuminuric pathway. In virtually all the available studies, renal biopsy was in fact performed for diagnostic purposes, i.e., in the presence of features raising suspicion of a non-diabetic renal disease such as glomerulonephritis, which was in fact highly prevalent, either isolated or in combination with diabetic nephropathy, as shown by a pooled meta-analysis of 48 studies including 4678 diabetic individuals, mainly with T2D [158]. In addition to exhibiting an atypical presentation and/or course of renal disease, virtually all patients included in these studies had albuminuria and most of them were proteinuric; therefore, no conclusion can be drawn regarding the anatomic substrate of nonalbuminuric renal impairment and research biopsy studies specifically focused on this phenotype are therefore required [159]. The only available study with these characteristics reported on renal biopsies from 31 T2D patients with reduced eGFR and either normoalbuminuria (n = 6, 19.4%), microalbuminuria (n = 8, 25.8%) or macroalbuminuria (n = 17, 54.8%). Results showed that individuals with micro/macro albuminuria had typical glomerular lesions, whereas half of those with normoalbuminuria showed atypical (vascular and/or tubulo-interstitial) or no lesions, but the other half still presented with diabetic glomerulosclerosis, though associated with varying degrees of arteriosclerosis [160]. Another study including 260 Japanese T2D patients with biopsy-proven diabetic nephropathy showed that glomerular lesions were associated with albuminuria, whereas glomerular, tubulo-interstitial, and vascular lesions were associated with reduced eGFR and were more advanced in individuals with normoalbuminuria and impaired renal function than in those with normoalbuminuria and preserved eGFR [161]. In addition, among patients with reduced eGFR, those with normoalbuminuria showed tubulo-interstitial and vascular lesions similar to or more advanced than glomerular lesions, compared with those with micro or macroalbuminuria [161]. However, a wide heterogeneity of renal lesions was observed also in a previous study on 34 microalbuminuric T2D patients with preserved eGFR, with 10 individuals (29.4%) showing no lesions, 10 (29.4%) showing typical glomerular lesions, and 14 (41.2) showing vascular and/or tubulo-interstitial lesions; interestingly, both HbA_1c_ levels and prevalence of retinopathy were higher in those with typical lesions [162]. Thus, atypical histological features are not specific of nonalbuminuric renal impairment, though probably more frequent in patients presenting with this phenotype, and vice versa typical lesions are not specific of the albuminuric form. Indeed, the Cohen rat, a T2D animal model of nonalbuminuric renal disease, shows only typical glomerular lesions [163]. Moreover, classical glomerulopathy can be detected in virtually all T1D patients with more than 5-year duration [164] and, in a more severe form, among those with normoalbuminuria and reduced eGFR [165]. No biopsy data are available from individuals showing early and rapid progressive renal decline, except for the finding that, in a small sample of Chinese T2D patients with renal biopsy, accelerated eGFR decline was predominantly associated with diabetic glomerulosclerosis [55].

Thus, at present, the clinical phenotype cannot be related to a specific anatomical phenotype, with presence or absence of albuminuria corresponding to typical glomerular and atypical vascular and/or tubulo-interstitial lesions, respectively. However, regardless of the anatomical substrate of the new phenotypes, the heterogeneity in the clinical presentation and course of DKD has important implications for the diagnosis, prognosis, and possibly treatment of this complication.

Box 1.4 It has been hypothesized that the nonalbuminuric phenotype associated with atypical vascular and/or tubulo-interstitial lesions, instead of the typical glomerular lesions. Unfortunately, there are no or insufficient renal biopsy data to confirm this hypothesis, though the available data indicate a wide heterogeneity of anatomical features in patients with T2D, but not in those with T1D, who almost invariably present with the classical glomerular lesions. Research biopsy studies specifically focused on the nonalbuminuric phenotype are therefore required.

### Diagnostic, prognostic and therapeutic implications

Current guidelines recommend to assess both albuminuria and eGFR for the screening of DKD [5]. Albuminuria should be measured preferably as urinary albumin-to-creatinine ratio (UACR) in a spot urine sample [56], in the absence of symptoms and signs of urinary tract infection or other interfering clinical conditions [56]. Assessment of urinary albumin excretion rate (UAER) in timed or 24-h collections is more troublesome but not more accurate than UACR, whereas measurement of albumin concentration in spot urine samples without simultaneously measuring urine creatinine is less expensive but also less accurate. Because of biological variability in albuminuria, two of three specimens of UACR (or UAER) collected within a 3- to 6-month period should be abnormal before considering a patient to have albuminuria, though in T2D individuals from the RIACE cohort concordance rate between the first value and the geometric mean of two-to-three measurements was > 90% for all albuminuria categories [166]. eGFR should be calculated from serum creatinine using a validated formula, preferably the CKD-EPI equation [56]. The emergence of the progressive renal decline phenotype suggests the importance of monitoring changes of eGFR over time to identify individuals experiencing an eGFR loss when their renal function is still within the normal range. To this end, though cystatin C-based eGFR [167] or cystatin C- and creatinine-based eGFR [168] may be preferable, serial measurements of serum creatinine may be sufficient, provided that they are frequent (at least once a year) and extend over a period of 3–5 years [52].

The diagnosis of DKD is usually made clinically, based on the presence of albuminuria and/or reduced eGFR, consistent with the finding that absence of albuminuria is a common feature in diabetic individuals with renal dysfunction. Currently, a renal biopsy for diagnostic purposes is indicated in case of atypical presentations that suggest the presence of other renal disorders which may benefit from specific treatment. Clinical features which raise suspicion of a non-diabetic renal disease include acute onset of proteinuria or rapid worsening of renal function, diabetes duration < 5 years (only for T1D patients), absence of retinopathy (which however is frequently lacking also in T2D individuals with DKD, especially in those without albuminuria), presence of active urine sediment (red or white blood cells or cellular casts), and symptoms or signs of other systemic diseases [3]. Though the real prevalence of non-diabetic renal disease in diabetic individuals is probably < 10% [1], this possibility should be always considered and a renal biopsy should be performed in the presence of the criteria listed above [159]. Conversely, a renal biopsy in patients with nonalbuminuric DKD is not indicated at present, though studies are urgently required for research purposes to understand the anatomical bases of this increasingly common phenotype [159].

Based on the level of albuminuria and eGFR, patients should then be assigned to the corresponding risk category according to the KDIGO CKD classification, which serves as a guide for frequency of monitoring and indicates the risk of progression to ESKD, but also of CVD events [56]. In fact, it has long been recognized that CKD from any cause is associated with a two-to-four-fold increased risk of morbidity and mortality from CVD since its early pre-dialytic stages, independent of traditional CVD risk factors [169]. In both T1D [170, 171] and T2D [23, 172], DKD represents a major contributor to excess all-cause and CVD death, possibly as a mediator of the relationship between hyperglycemia and adverse outcomes. While DKD-related mortality risk is much higher in younger individuals, DKD appears to fully account for excess risk of death associated with T2D only in older patients [172, 173]. Both albuminuria and reduced eGFR were shown to be associated with all-cause and CVD mortality, independently of each other, both in the general population [174–177] and in patients with T1D [171, 178] and T2D [42, 172, 173].

Recent reports have examined the mortality risk associated with the different DKD phenotypes in patients with T2D. A post hoc analysis of the ADVANCE Study (10,640 T2D participants) showed that risk of CVD death associated with nonalbuminuric DKD was similar to that of microalbuminuria with an eGFR ≥ 90 ml/min/1.73 m^2^, but lower than that of microalbuminuria with an eGFR 60–89 ml/min/1.73 m^2^ and of macroalbuminuria with an eGFR > 60 ml/min/1.73 m^2^ [42]. Conversely, *a* post hoc analysis of the FIELD Study (9795 T2D participants) showed that the nonalbuminuric phenotype was associated with a higher risk of death from CVD, non-CVD, and any cause, compared with microalbuminuria with an eGFR > 60 ml/min/1.73 m^2^ and macroalbuminuria with an eGFR ≥ 90 ml/min/1.73 m^2^ [41]. However, due to the selection criteria for trial entry, only a limited number of individuals with an eGFR < 60 ml/min/1.73 m^2^ were enrolled in these two studies. The community-based Casale Monferrato Study (1565 patients with T2D) reported a significant association between reduced eGFR and mortality only among macroalbuminuric individuals [179]. In contrast, data from the NHANES 1988–1994 (1430 diabetic individuals) showed that the standardized 10-year mortality among patients with the nonalbuminuric phenotype was intermediate between the albuminuric DKD phenotypes with preserved and reduced eGFR [23]. In the Cardiovascular Health Study (691 older diabetic adults), the adjusted risk of death was similar for albuminuria alone and reduced eGFR alone [180]. Likewise, data from the RIACE cohort (15,773 T2D patients) showed that risk of death of reduced eGFR alone was similar to that of albuminuria alone. Moreover, in normoalbuminuric patients with an eGFR 45–59 ml/min/1.73 m^2^, risk was similar to that of patients with microalbuminuria alone and, in those with an eGFR < 45 ml/min/1.73 m^2^, risk was similar to that of patients with macroalbuminuria alone [181]. Finally, a recent analysis of the NHANES 2003–2006 data showed that age-standardized mortality risk for nonalbuminuric DKD was lower than for macroalbuminuria with eGFR 60–89 60 ml/min/1.73 m^2^, but higher than for microalbuminuria alone and macroalbuminuria with eGFR ≥ 90 ml/min/1.73 m^2^ [75]. Noteworthy, this analysis also showed that mortality rates in adults with diabetes have decreased among individuals with increased albuminuria and increased in those with decreased eGFR and normoalbuminuria from 1988 to 2006 [75]. These diverging temporal trends in mortality might also explain, at least partly, the differences in the risk of death associated with isolated albuminuria and reduced eGFR among the above studies. Similar findings have been reported in patients with T1D. In the FinnDiane Study, the nonalbuminuric phenotype was associated with an increased risk of CVD and all-cause mortality to the same extent as individuals with albuminuria alone [45]. Likewise, in a study from Tuscany, the risk of all-cause death associated with reduced eGFR alone was similar to that of increased albuminuria alone, with the highest mortality occurring in T1D patients with both reduced eGFR and albuminuria [182].

Regarding CVD events, data from the RIACE cohort have shown that the age- and gender-adjusted thresholds at which CVD burden increases in T2D individuals stand near to or within the normal range for both eGFR (78.2 ml/min/1.73 m^2^) and albuminuria (10.5 mg/24 h). Moreover, the prevalence of any CVD event was intermediate in the nonalbuminuric phenotype, i.e. higher than that of albuminuria alone and lower than that of combined albuminuria and reduced eGFR. Interestingly, coronary events correlated more strongly with the nonalbuminuric phenotype than with the albuminuric forms, whereas the opposite was observed for cerebrovascular and peripheral events [183]. The ADVANCE Study showed that, over a 4.3-year follow-up, the hazard ratio for CVD events was similar for reduced eGFR and albuminuria, whereas it was markedly higher when both abnormalities were present [42]. Regarding renal outcomes, the absence of albuminuria was found to be associated with a lower risk in patients with T2D from the CRIC Study [40] and the ADVANCE Study [42] and also in individuals with T1D from the FinnDiane Study [45]. Similar results were previously obtained in a small study showing that, over a 38-month follow-up, no normoalbuminuric patient with reduced eGFR died or developed ESKD, as opposed to 5 patients with microalbuminuria and 17 with macroalbuminuria [184]. Likewise, the analysis of a population-based district diabetes registry showed that the annual eGFR decline was 0.3% in normoalbuminuric, 1.5% in microalbuminuric, and 5.7% in macroalbuminuric patients with T1D and T2D and a mean eGFR > 75 ml/min/1.73 m^2^ [61].

Thus, though less prone to progress to ESKD, the nonalbuminuric phenotype appears to be associated with a significant risk of CVD morbidity and mortality, which is equal to or even higher than that associated with albuminuria alone and requires a higher level of attention and care than that generally provided.

Concerning therapeutic measures, the increasing prevalence of reduced eGFR [11, 12] and the increasing mortality associated with it, especially in the absence of albuminuria [75], indicate that changes in treatment, particularly the increasing use of RAS blockers, have not impacted favourably on eGFR decline and the nonalbuminuric phenotype. This implies that albuminuria and eGFR loss may require different therapeutic interventions and that treatments which are effective in slowing down eGFR decline are urgently required.

Thus, on purely theoretical grounds, use of angiotensin converting enzyme (ACE) inhibitors and angiotensin receptor blockers (ARBs) may not be indicated in individuals presenting with the nonalbuminuric phenotype, and may even be deleterious as these agents increase susceptibility to renal ischemia by preventing the rise of efferent arteriolar resistance [185]. Unfortunately, there are no data supporting this assumption, due to the lack of intervention trials specifically targeting individuals with the nonalbuminuric phenotype. So far, studies have in fact included almost exclusively patients with micro or macroalbuminuria in order to assess the efficacy of an intervention in favouring regression or blocking progression of albuminuria [9]. In trials with RAS blockers, ACE inhibitors and ARBs were shown to provide similar benefits [186, 187] and to be effective beyond their blood pressure-lowering effect in preventing progression to ESKD in patients with macroalbuminuria [80–82], but not in the setting of lower levels of albuminuria [188, 189].

Box 1.5 Diagnosis of DKD is based on both albuminuria and eGFR, preferably calculated using the CKD-EPI equation. Albuminuria should be confirmed in two of three urine specimens collected within a 3- to 6-month period, whereas the slope of eGFR should be calculated from frequent measurements of serum creatinine and/or cystatin C, starting when renal function is normal. A renal biopsy should be performed when there is suspicion of a non-diabetic renal disease. Prognosis of DKD is influenced by the increased risk of progression toward ESKD as well as of morbidity and mortality from CVD. Compared with the classical albuminuric phenotype, the nonalbuminuric phenotype is associated with an equal CVD risk, whereas risk of progression to ESKD is lower. Treatment of DKD is effective in reducing albuminuria, but not eGFR decline, suggesting that these two DKD manifestations may require different therapeutic strategies, though there are no data from clinical trials on individuals with nonalbuminuric renal impairment or progressive renal decline.

## Treatment of hyperglycemia in T2D patients with impaired renal function

Treatment of hyperglycemia in patients with T2D and impaired renal function represents a major challenge for a number of reasons, which may impose avoidance/discontinuation or dose adjustment of certain anti-hyperglycemic drugs. First, together with the liver, the kidney is a major site for drug metabolism and excretion [190]. This implies that circulating levels of agents that are degraded and/or eliminated via the renal route may increase in these individuals, thus enhancing the risk of adverse effects including hypoglycemia. Second, impaired renal function per se is a risk factor for hypoglycemia, even in non-diabetic individuals [191], as the kidney contributes to total endogenous glucose production by approximately 30% [192]. In addition, in individuals with impaired renal function, hypoglycemia is favored by the coexistence of acidosis, which limits the ability of the liver to compensate for reduced renal gluconeogenesis [193] as well as of malnutrition and/or muscle wasting, which decrease hepatic glycogen stores and the availability of gluconeogenic substrates [194]. Third, as patients with impaired renal function are usually excluded from clinical trials, evidence on the efficacy and safety of several anti-hyperglycemic agents is lacking in these individuals, especially in those with an eGFR < 30 ml/min/1.73 m^2^ [195]. Finally, as compared with patients without DKD, those with DKD are usually older, with longer diabetes duration, more frequently suffering from comorbidities, especially CVD, [196] and, hence, on multiple medications with potential interactions with anti-hyperglycemic drugs [197].

Nevertheless, the therapeutic options for T2D individuals with impaired renal function have substantially increased over the last decades. On the one hand, several new classes of anti-hyperglycemic drugs have been recently made available for treatment of T2D [198]. Of these, the glucagon-like peptide 1 (GLP-1) receptor agonists and the dipeptidyl peptidase 4 (DPP-4) inhibitors can be used safely in individuals with impaired renal function, whereas the use of the inhibitors of sodium-glucose cotransporter 2 (SGLT2) is limited [199]. In addition, these new agents do not cause hypoglycemia, except when used in combination with insulin and/or insulin secretagogues, and, more importantly, cardiovascular outcome trials have shown that, along with cardiovascular benefits, GLP-1 receptor agonists and SGLT2 inhibitors provide also renal protection, thus opening promising perspectives for the prevention and treatment of DKD [200]. Of note, renal protection from GLP-1 receptor agonists was limited to reduced progression of albuminuria, whereas SGLT2 inhibitors appeared to slow down also the decline of eGFR, though renal outcomes were not primary endpoints in these trials [200]. On the other hand, recent real-world data have shown a widespread use of old drugs such as metformin and sulfonylureas in patients with reduced renal function, even beyond the current labeling contraindications [201]. Nevertheless, these data have also shown that, in these individuals, risk of lactic acidosis with metformin is lower than expected [202], thus prompting reconsideration of its use in patients with moderately reduced renal function, who would otherwise be excluded from the beneficial effects of this agent [5].

**Fig. 2:**
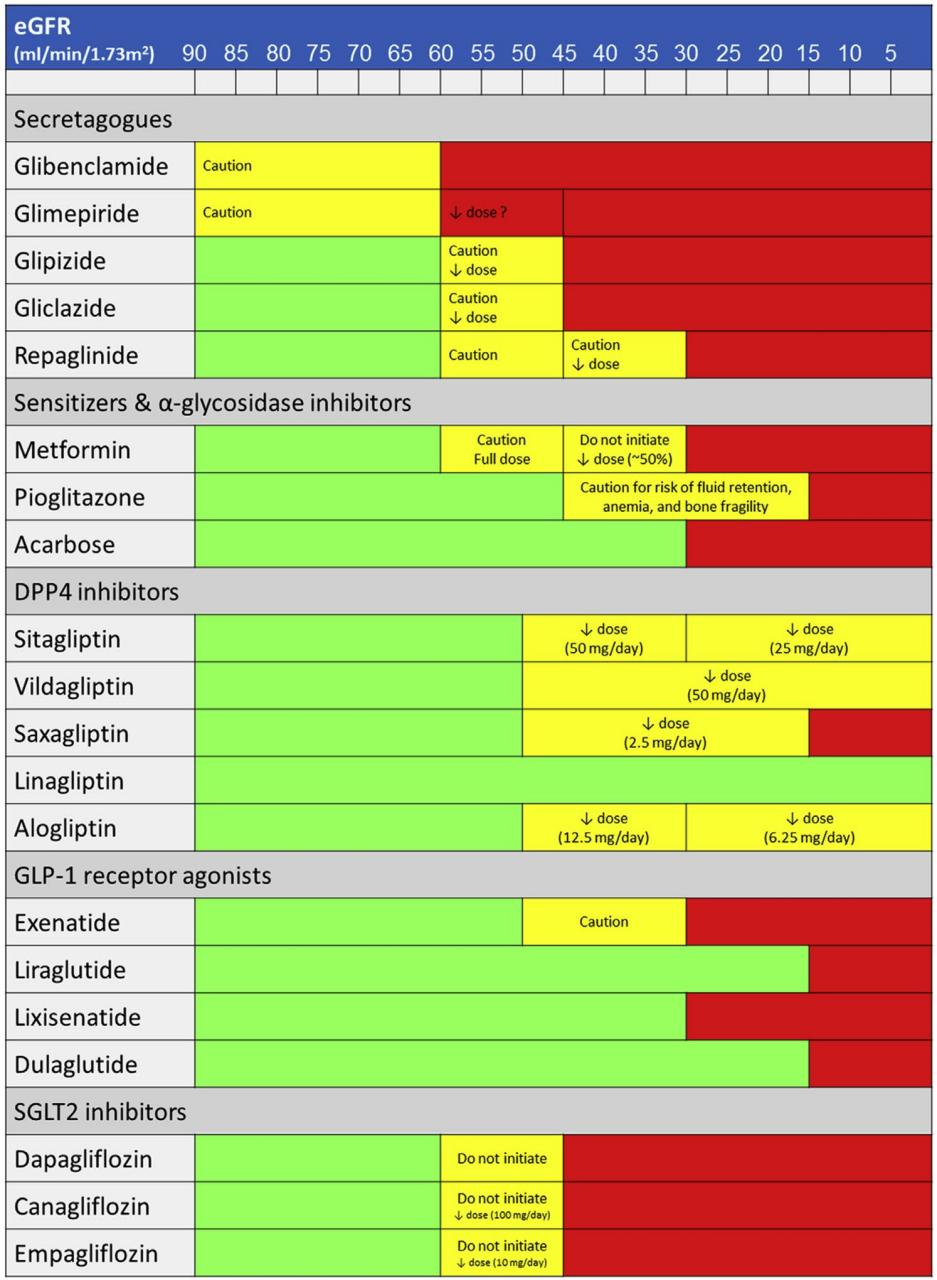
Recommended usage and dosage of currently available non-insulin drugs according to the level of eGFR. *eGFR* estimated glomerular filtration rate, *DPP-4* dipeptidyl peptidase 4, *GLP-1* glucagon-like peptide 1, *SGLT2* sodium-glucose cotransporter 2

Figure 2 shows the recommended usage and dosage of currently available non-insulin drugs according to the level of eGFR.

Box 2 Treatment of hyperglycemia in T2D patients with impaired renal function represents a major challenge for a number of reasons, which may impose avoidance/discontinuation or dose adjustment of certain anti-hyperglycemic drugs. The therapeutic options for T2D patients with impaired renal function have substantially increased over the last decades, due to the availability of several new classes of anti-hyperglycemic drugs, which do not cause hypoglycemia and, in some cases, seem to provide cardiorenal protection, and to the reconsideration of the use of old drugs such as metformin in these individuals.

### Insulin and insulin secretagogues

Due to the increased risk of hypoglycemia associated with renal dysfunction, insulin and insulin secretagogues should be used with caution in patients with reduced eGFR.

Nevertheless, insulin treatment with both human preparations and insulin analogs is safe in all eGFR categories, though it may be necessary to reduce the dosage in patients with advanced renal dysfunction, especially for human insulins, which are metabolized by insulinase in both the liver and kidney [203]. The reduction in insulin clearance has been estimated to range between 10 and 20% in patients with moderate-to-severe CKD [204].

Conversely, the use of the insulin secretagogues sulfonylureas and meglitinides, which stimulate insulin release by the β-cell in a glucose-independent manner [205], should be limited in patients with impaired renal function, though to a various extent depending on the specific compound. Glibenclamide (also known as glyburide) should be avoided in patients with any degree of renal impairment [5, 199, 206], because of its long duration of action and the renal excretion of active metabolites resulting from hepatic metabolism of the drug [207]. For the same reasons [208], glimepiride should be avoided or initiated conservatively at 1 mg daily in patients with reduced eGFR [5, 199, 206]. Gliclazide and glipizide are not contraindicated in patients with renal dysfunction, since they are metabolized by the liver and excreted in the urine as inactive metabolites [209, 210]; however, caution is recommended also for these agents [5, 199, 206] and glipizide should be initiated conservatively at 2.5 mg daily in patients with reduced eGFR [5]. Finally, the meglitinide repaglinide is a short-acting secretagogue that is also metabolized by the liver to inactive metabolites, which are excreted via the bile into the feces [211]. For these reasons, repaglinide is largely utilized across all eGFR categories, despite the increased risk of hypoglycemia, which becomes relevant for low levels of eGFR (< 30 ml/min/1.73 m^2^). Therefore, repaglinide should be initiated conservatively at 0.5 mg [5, 199, 206] and the dose should be adjusted or the drug substituted with a safer agent such as a DPP-4 inhibitor in case of declining eGFR.

Box 2.1 Insulin treatment with both human preparations and insulin analogs is safe in all eGFR categories, though it may be necessary to reduce the dosage in patients with advanced renal dysfunction. The use of the insulin secretagogues sulfonylureas and meglitinides should be limited in patients with impaired renal function because of the increased risk of hypoglycemia. Glibenclamide should be avoided, glimepiride should be avoided or initiated conservatively at 1 mg daily, and gliclazide, glipizide, and repaglinide should be used with caution at reduced dose.

### Insulin sensitizers and inhibitors of α-glycosidase

The insulin sensitizers, biguanides and thiazolidinediones, and the inhibitors of α-glycosidase are associated with low risk of hypoglycemia.

Among the insulin sensitizers, the biguanide metformin is the first-line drug for the treatment of T2D [5], albeit its mechanism of action is still debated [212], with an increasingly recognized effect at the gut level in addition to that in the liver [213]. As metformin is not metabolized by the liver and is excreted unchanged by the kidney [214], its plasma concentrations rise in patients with renal impairment; therefore, it is contraindicated in these individuals, though the eGFR threshold has been lowered to < 30 ml/min/1.73 m^2^ [5, 199, 206, 215]. Moreover, metformin should be used at reduced dose (by approximately 50%) or it should not be started in patients with an eGFR 30–45 ml/min/1.73 m^2^, whereas no dose adjustment is required for an eGFR > 45 ml/min/1.73 m^2^ [5, 199, 206, 215]. Pending the results of ongoing clinical trials, even less stringent eGFR thresholds might be recommended for delayed-release metformin preparations that target the ileum, which minimize systemic exposure while maintaining glucose-lowering efficacy [216]. Conversely, conditions characterized by lactate overproduction from hypoxic tissues, as in respiratory and circulatory failure and severe anemia, and/or impaired lactate removal due to impaired gluconeogenesis, as in advanced liver disease, may precipitate lactic acidosis in individuals with reduced renal function treated with metformin and, hence, require drug discontinuation [216].

Pioglitazone, the only thiazolidinedione compound currently available for clinical use in most European countries, activates peroxisome proliferator-activated receptor γ, a nuclear receptor regulating the transcription of genes involved in glucose and lipid metabolism, thus increasing insulin sensitivity [217]. It is metabolized entirely by the liver [218] and, hence, no dose adjustment is required according to the level of eGFR [5, 199, 206]. However, caution is recommended in patients with advanced renal dysfunction, due to the increased risk of fluid retention, anemia, and bone fragility characterizing these individuals, which may be enhanced by the use of pioglitazone [5, 199, 206].

Acarbose is an inhibitor of α-glycosidase that splits polysaccharides into monosaccharides, thus delaying intestinal glucose absorption and reducing post-prandial glycaemia [219]. It is metabolized by intestinal bacteria, with production of several metabolites, at least one of which has some biological activity; however, only a small amount of the drug is absorbed [220] and less than 2% is recovered in the urine as an active drug, either intact compound or active metabolite [221]. For this reason and for the limited evidence in patients with severe renal insufficiency, acarbose should be avoided in individuals with an eGFR < 30 ml/min/1.73 m^2^ [5, 199, 206].

Box 2.2 Metformin is contraindicated in patients with an eGFR < 30 ml/min/1.73 m^2^ and in conditions characterized by lactate overproduction from hypoxic tissue and/or impaired lactate removal. It should be used at reduced dose (by approximately 50%) or should not be started in individuals with an eGFR 30–45 ml/min/1.73 m^2^. Pioglitazone can be used without dose adjustment, though caution is recommended in patients with advanced renal dysfunction, due to the increased risk of fluid retention, anemia, and bone disease. Acarbose should be avoided in individuals with an eGFR < 30 ml/min/1.73 m^2^.

### Incretin mimetics

The incretin mimetics include the DPP4 inhibitors, which block the DPP4-mediated breakdown of the incretins GLP-1 and gastric inhibitory polypeptide (GIP), thus increasing and maintaining endogenous GLP-1 and GIP levels, and the GLP-1 receptor agonists, which are DPP4-resistant incretin analogues derived from exendin-4 or human GLP-1 [222]. By increasing endogenous or exogenous incretin levels, these agents stimulate insulin and inhibit glucagon secretion in a glucose-dependent manner, thus reducing blood glucose levels without causing hypoglycemia [222]. In addition, by virtue of the pharmacological incretin levels achieved with GLP-1 receptor agonists, these agents reduce appetite by delaying gastric emptying and inhibiting hypothalamic orexigenic signaling and, hence, produce body weight loss [223].

All the DPP-4 inhibitors are metabolized by the liver, though to a different extent, and are excreted by the kidney, with the exception of linagliptin, only ~ 5% of which is found in the urines [224]. Therefore, while dosage of sitagliptin, vildagliptin, saxagliptin, and alogliptin should be reduced according to the level of eGFR, linagliptin requires no dose adjustment [5, 199, 206]. However, all the DPP-4 inhibitors can be used safely in patients with renal dysfunction and, except saxagliptin, even in those on dialysis [5, 199, 206]. The excellent safety profile of these agents, including the very low risk of hypoglycemia, makes them the first treatment option in elderly patients with reduced renal function and mild-to-moderate metabolic derangement who do not require specific cardiovascular protection [225]. In these individuals, they should be preferred to secretagogues, including repaglinide.

Among the GLP-1 receptor agonists, only the exendin-4-derived exenatide and lixisenatide are excreted by the kidney and, hence, these agents should be avoided if eGFR is < 30 ml/min/1.73 m^2^. Conversely, the human GLP-1-derived liraglutide and dulaglutide can be used up to an eGFR of 15 ml/min/1.73 m^2^, whereas there is insufficient experience with these agents for lower eGFR values [226]. Use of these agents may be associated with gastrointestinal symptoms, which however tend to disappear with time [227]. Due to their robust glucose-lowering activity, they represent an effective and safe alternative to insulin or, in combination with basal insulin, to basal-bolus regimens, to reduce the risk of hypoglycemia and body weight gain [225]. In addition, they are a first-line treatment in obese patients and in those with atherosclerotic cardiovascular disease because of their cardiovascular benefits [225]. In order to provide renal protection, they may be used also in patients with albuminuria and an eGFR < 60 or 45 ml/min/1.73 m^2^ as an alternative to SGLT2 inhibitors [225].

Box 2.3 The DPP-4 inhibitors can be used in patients with impaired renal function, albeit at reduced dosage (except for linagliptin, which does not require dose adjustment), are weight neutral and have an excellent safety profile. The GLP-1 receptor agonists can be used up to an eGFR of 30 ml/min/1.73 m^2^ (exenatide and lixisenatide) or 15 ml/min/1.73 m^2^ (liraglutide and dulaglutide), favor weight loss and provide protection from cardiovascular and renal disease (the latter limited to albuminuria), but their use may be associated with gastro-intestinal symptoms.

### SGLT2 inhibitors

The SGLT2 inhibitors act at the kidney level by inhibiting glucose (and sodium) reabsorption in the proximal tubule, thus causing glycosuria, osmotic diuresis and, at least initially, natriuresis [228]. Energy loss with glycosuria produces weight loss, whereas water loss with diuresis results in volume depletion and reduction of blood pressure [228]. Adverse effects include genital and urinary tract infections, symptoms of volume depletion, and euglycemic ketoacidosis [229]. As glucose reabsorption by the proximal tubule is linearly related to blood glucose levels and glucose filtration by the glomerulus, the SGLT2 inhibitors do not cause hypoglycemia, but display insufficient glucose-lowering effect in individuals with reduced eGFR [230]. Therefore, these agents should not be initiated or should be discontinued for an eGFR < 60 or 45 ml/min/1.73 m^2^, respectively; in addition, dose of empagliflozin and canagliflozin should be reduced at 10 and 100 mg daily, respectively, if eGFR is 45-59 ml/min/1.73 m^2^ [5, 199, 206]. Though less potent than GLP-1 receptor agonists, the SGLT2 inhibitors may be used to reduce insulin requirements and the risk of hypoglycemia in insulin-treated patients [228]. More importantly, they are indicated in obese patients and represent a first-choice treatment option in those with atherosclerotic cardiovascular disease, chronic heart failure, and/or DKD, provided that eGFR is adequate [228]. Since cardiovascular outcome trials with SGLT2 inhibitors showed that cardiorenal protection and the blood pressure (and body weight) reducing effects were maintained in patients with an eGFR < 60 ml/min/1.73 m^2^ [231], current eGFR limits for use of these agents might be reconsidered in the future. The positive results of a recent clinical trial conducted in CKD individuals with renal outcomes as primary endpoints [232] provide further support to this concept.

Box 2.4 The SGLT2 inhibitors can be used up to an eGFR of 45 ml/min/1.73 m^2^, as they display insufficient glucose-lowering effect below this level, favor weight loss and provide protection from cardiovascular and renal disease (the latter extended to eGFR loss), but their use may be associated with side effects.

### Additional considerations

The above mentioned eGFR thresholds below which some anti-hyperglycemic agents should be used at reduced dosage or even discontinued imply that renal function should be regularly monitored in patients with T2D, at intervals depending on the actual eGFR level and its stability over time. In addition, patients should be advised to stop the medication in cases of dehydration, which may abruptly reduce eGFR and increase the risk of drug side effects. This is particularly important for treatment with agents favoring dehydration by causing gastrointestinal symptoms such as nausea, vomiting, and diarrhea, i.e., metformin, acarbose, and GLP-1 receptor agonists, or increased diuresis, such as the SGLT2 inhibitors. In case of eGFR instability over time, anti-hyperglycemic agents which need dose adjustment or discontinuation below certain eGFR thresholds should not be used.

Box 2.5 Treatment of patients with impaired renal function with anti-hyperglycemic agents which need dose adjustment or discontinuation below certain eGFR thresholds require regular eGFR monitoring. In case of instability of eGFR over time these agents should not be used.

## Conclusions

During the last decades, the unique heterogeneity of the natural history of DKD has progressively emerged, possibly as a result of improved treatment. In particular, two new phenotypes, nonalbuminuric renal impairment and progressive renal decline, have been described. However, though these phenotypes have been increasingly recognized, their pathogenesis and anatomical correlates are still unclear and require further investigation and performing of research biopsy studies.

In the same time period, several new classes of anti-hyperglycemic drugs have been made available for treatment of T2D patients, including those with impaired renal function, and some of these agents have shown cardiorenal protection. In addition, the use of certain old agents in patients with impaired eGFR has been reconsidered, thus further increasing the therapeutic options in these individuals.
